# A Novel Dimeric Inhibitor Targeting Beta2GPI in Beta2GPI/Antibody Complexes Implicated in Antiphospholipid Syndrome

**DOI:** 10.1371/journal.pone.0015345

**Published:** 2010-12-15

**Authors:** Alexey Kolyada, Chang-Jin Lee, Alfredo De Biasio, Natalia Beglova

**Affiliations:** Department of Medicine, Beth Israel Deaconess Medical Center and Harvard Medical School, Boston, Massachusetts, United States of America; University of South Florida College of Medicine, United States of America

## Abstract

**Background:**

β2GPI is a major antigen for autoantibodies associated with antiphospholipid syndrome (APS), an autoimmune disease characterized by thrombosis and recurrent pregnancy loss. Only the dimeric form of β2GPI generated by anti-β2GPI antibodies is pathologically important, in contrast to monomeric β2GPI which is abundant in plasma.

**Principal Findings:**

We created a dimeric inhibitor, A1-A1, to selectively target β2GPI in β2GPI/antibody complexes. To make this inhibitor, we isolated the first ligand-binding module from ApoER2 (A1) and connected two A1 modules with a flexible linker. A1-A1 interferes with two pathologically important interactions in APS, the binding of β2GPI/antibody complexes with anionic phospholipids and ApoER2. We compared the efficiency of A1-A1 to monomeric A1 for inhibition of the binding of β2GPI/antibody complexes to anionic phospholipids. We tested the inhibition of β2GPI present in human serum, β2GPI purified from human plasma and the individual domain V of β2GPI. We demonstrated that when β2GPI/antibody complexes are formed, A1-A1 is much more effective than A1 in inhibition of the binding of β2GPI to cardiolipin, regardless of the source of β2GPI. Similarly, A1-A1 strongly inhibits the binding of dimerized domain V of β2GPI to cardiolipin compared to the monomeric A1 inhibitor. In the absence of anti-β2GPI antibodies, both A1-A1 and A1 only weakly inhibit the binding of pathologically inactive monomeric β2GPI to cardiolipin.

**Conclusions:**

Our results suggest that the approach of using a dimeric inhibitor to block β2GPI in the pathological multivalent β2GPI/antibody complexes holds significant promise. The novel inhibitor A1-A1 may be a starting point in the development of an effective therapeutic for antiphospholipid syndrome.

## Introduction

Beta2-glycoprotein I (β2GPI) is the major target for autoimmune antibodies associated with antiphospholipid syndrome (APS), an autoimmune disease characterized clinically by thrombosis and recurrent pregnancy loss [1], [2], [3], [4]. Presently, APS patients with thrombotic complications who have high titers of antibodies are treated chronically with anticoagulants [5], [6], [7]. However, even continuous anticoagulation may not prevent recurrent thrombosis [5], emphasizing the need for a more effective antithrombotic therapy based on the thrombogenic mechanisms specific to APS.

β2GPI consists of five domains [8], [9]. Flexible linkers between domains permit β2GPI to adopt different overall shapes such as a fishhook-like shape seen in the crystal structure [8], [9], an S-shape observed by small angle x-ray scattering for β2GPI in solution [10] and a circular shape detected by electron microscopy [11]. The circular shape in which domain I is adjacent to domain V is the predominant conformation of β2GPI in normal human plasma [11]. Circular β2GPI can be converted to an extended form by altering pH and salt concentrations, binding to a high-affinity antibody directed to domain I or by the binding to cardiolipin [11]. β2GPI, which is abundant in plasma (about 170 µg/ml or 4 µM) [12], acquires its prothrombotic properties only in the presence of anti-β2GPI antibodies. Antibodies of the IgG isotype have the highest correlation with the clinical manifestations of APS compared to other identified antibodies [13], [14]. Although anti-β2GPI antibodies in APS patients are highly heterogeneous in respect to their affinity for β2GPI and the location of their binding epitopes, autoantibodies against domain I are the most common and better correlate with thrombosis [15], [16]. The presence of anti-β2GPI antibodies causes cellular activation both in vitro and in vivo [17], [18], [19]. Toll-like receptors, annexin A2, ApoE receptor (ApoER2), platelet receptor GPIb and anionic phospholipids exposed on cellular surfaces of activated cells are suggested to be pathologically important in APS [18], [19], [20], [21], [22], [23], [24], [25], [26], [27], [28], [29]. The binding sites for anionic phospholipids [30], [31], [32], [33], lipoprotein receptors (including ApoER2) [34] and GPIb [21] are in domain V of β2GPI (β2GPI-DV).

In the present studies, we are suggesting a novel approach to interference with anti-β2GPI-dependent thrombosis in APS. To prevent the β2GPI/antibody complexes from the binding to receptors, we designed an inhibitor that a) targets β2GPI and b) binds tightly to β2GPI/antibody complexes expressing the dimeric β2GPI but binds weakly to β2GPI monomers. These requirements have the following rationale: First, complete β2GPI deficiency in humans, although rare, does not lead to apparent health problems [35], [36], [37], therefore the inhibitor that targets β2GPI will not disrupt normal biological processes. Second, β2GPI/anti-β2GPI antibody complexes expressing dimeric β2GPI but not monomeric β2GPI are pathologically important [28], [38], therefore the inhibitor should bind preferentially to β2GPI/anti-β2GPI complex compared to β2GPI monomers. Anti-β2GPI antibodies constitute less than 3% of total IgG in patients with antiphospholipid syndrome and have weak affinity for β2GPI [39], [40], [41]. In contrast to β2GPI monomers which are abundant in plasma, β2GPI/anti-β2GPI complexes are present at low concentration.

In this study, we are focusing on the inhibition of the binding of β2GPI/anti-β2GPI antibody complexes to ApoER2 and to anionic phospholipids. ApoER2, like other members of the family of lipoprotein receptors, binds β2GPI via structurally homologous ligand-binding type A (LA) modules and the first LA module of ApoER2 is the most important for the binding [42], [43], [44], [45]. Recently, we have shown that different LA modules bind to the same site on β2GPI and that β2GPI can not simultaneously bind an LA module and a cardiolipin-coated surface [46]. Therefore, an LA module bound to β2GPI has dual action: it inhibits both the binding of β2GPI to lipoprotein receptors and anionic phospholipids expressed on cells.

We created a dimeric inhibitor, A1-A1. To make this inhibitor, we isolated the first LA module from ApoER2 (A1) and connected two A1 modules with a flexible linker. In the present studies, we compared a monomeric A1 with A1-A1 on interfering with the binding of β2GPI/anti-β2GPI antibody complexes to anionic phospholipids. We tested the inhibition of β2GPI present in human serum, β2GPI purified from human plasma and domain V of β2GPI. β2GPI in serum is the circular form of β2GPI [11] and the individual domain V represents β2GPI in the extended conformation. We demonstrated that when β2GPI/antibody complexes are formed, A1-A1 is more effective than A1 in inhibition of β2GPI binding to cardiolipin, regardless of the source of β2GPI. Similarly, A1-A1 strongly inhibits the binding of dimerized domain V of β2GPI to cardiolipin compared to the monomeric A1 inhibitor. Moreover, A1-A1 preferentially binds β2GPI/anti-β2GPI antibody complexes and binds only weakly to monomeric β2GPI. The novel inhibitor A1-A1 may be a starting point in the development of an effective drug for prevention and treatment of β2GPI-dependent thrombosis in antiphospholipid syndrome.

## Results

### Design of a dimeric inhibitor

**Figure 1:**
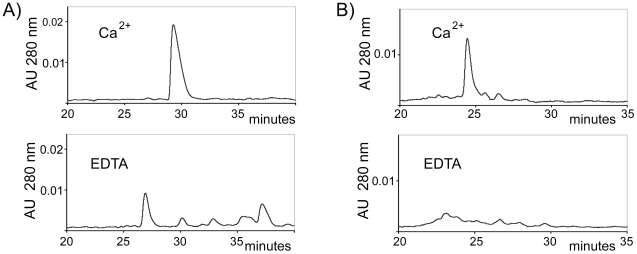
HPLC chromatograms of products formed by oxidative refolding of A1 (A) and A1-A1 (B). The proteins were dialyzed in redox buffer in the presence of calcium or EDTA and eluted with a linear gradient of 0.1% per minute of acetonitrile containing 0.1% TFA starting at 15 minutes from 21% of acetonitrile/TFA (for A1) or 26% of acetonitrile/TFA (for A1-A1).

Recently, we have shown that the first LA module from ApoER2 (A1) binds domain V of β2GPI with 1 µM affinity [46]. In order to target a multivalent β2GPI formed by anti-β2GPI antibodies, we made a dimeric inhibitor consisting of two A1 modules covalently connected by a linker. To allow largely unrestricted relative motion of two A1 modules in the A1-A1 molecule, we used a flexible linker Gly-Ser-Ser-Gly to connect A1 modules. In extended conformation, this four-residue linker is capable of separating the A1 modules in A1-A1 by up to 15 Å. We expressed and purified A1-A1 using the same procedure that we have previously used for the expression and purification of other LA modules including A1. Our previous analysis of different recombinantly expressed LA modules by solution NMR spectroscopy and crystallography demonstrated that the purified LA modules are properly folded, their structures are identical to the structures of these modules in full-length receptors and, in the presence of calcium, recombinant LA modules bind their ligands β2GPI, β2GPI-DV, RAP and ApoE [46], [47], [48], [49]. Because calcium is essential for the function of LA modules and the formation of native disulfide bonds [50], we analyzed the folding of the dimeric molecule, A1-A1, in the presence and absence of calcium. We compared the oxidative refolding of A1-A1 to the refolding of A1, which yields a functional A1 module. The same quantities of the recombinant proteins were dialyzed in redox buffer containing either calcium or EDTA. After 36 hours (samples with A1) or 72 hours (samples with A1-A1) of refolding, the proteins were acidified with 0.1% TFA to stop the disulfide exchange and analyzed by a reversed-phase HPLC on an analytical C18 column. The A1 module contains six cysteine residues forming three disulfide bonds. In the presence of calcium, both A1 and A1-A1 converged to unique disulfide-bonded species out of many possibilities providing evidence that the presence of calcium guided formation of native disulfide bonds (Figure 1A,B). For comparison, refolding of A1 and A1-A1 in the presence of EDTA yielded a distribution of multiple disulfide-bonded isomers.

### Comparison of the dimeric inhibitor A1-A1 with monomeric A1. Inhibition of the binding of β2GPI to cardiolipin

**Figure 2:**
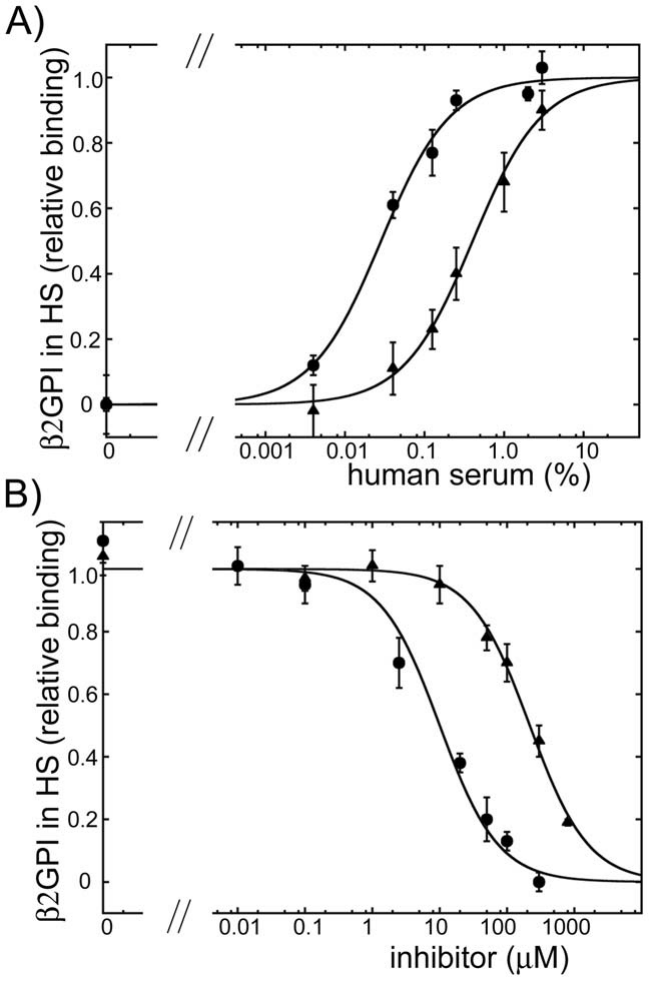
Binding and inhibition of the binding of β2GPI in normal human serum to cardiolipin. A) The binding of β2GPI in serum to cardiolipin-coated surface in the absence (triangles) and in the presence (circles) of anti-β2GPI antibodies. B) Inhibition of the binding of β2GPI in human serum to cardiolipin in the presence of anti-β2GPI antibodies by the dimeric inhibitor A1-A1 (circles) and monomeric inhibitor A1 (triangles). Fit to one-site binding and inhibition models was performed on the raw data. To facilitate comparison, the measured OD_405_ values and the binding curves were normalized to the maximum binding obtained from the fit.

**Figure 3:**
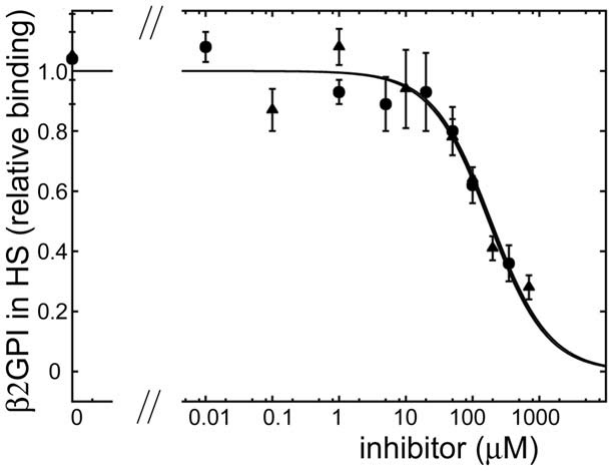
Inhibition of the binding of β2GPI in human serum to cardiolipin in the absence of anti-β2GPI antibodies. Inhibition curves measured for the dimeric inhibitor A1-A1 (circles) and monomeric inhibitor A1 (triangles). On the plot, the data points at 50 µM and 100 µM of A1-A1 partially overlap with the corresponding data points of A1. To facilitate comparison, the measured OD_405_ values and the binding curves were normalized to the maximum binding obtained from the fit of the raw data to a one-site inhibition model.

Anti-β2GPI antibodies create multivalent β2GPI/anti-β2GPI complexes that have pathological properties compared to pathologically inactive β2GPI monomers, which are normally present in plasma. The binding of β2GPI to anionic phospholipids in the presence of anti-β2GPI antibodies is one of the pathological mechanisms leading to thrombosis and pregnancy losses in antiphospholipid syndrome. We compared the dimeric inhibitor, A1-A1, to monomeric A1 on the inhibition of the binding of β2GPI/anti-β2GPI antibody complexes to cardiolipin coated on a plate. First, we used pooled normal human serum as a source of β2GPI. The majority of the β2GPI molecules in serum is in the circular form [11]. To select the appropriate concentration of β2GPI for the inhibition studies, we measured the binding curves (Figure 2A). To create β2GPI/anti-β2GPI complexes, anti-β2GPI antibodies at constant concentration were added to β2GPI before the samples were applied to cardiolipin. The presence of anti-β2GPI antibodies significantly enhanced the binding of β2GPI to cardiolipin reaching the half-maximal binding at 0.028±0.004% and 0.40±0.05% of serum in the presence and in the absence of anti-β2GPI antibodies, respectively. We then compared the efficiency of A1-A1 and A1 for the inhibition of the binding of β2GPI in serum to cardiolipin in the presence of anti-β2GPI antibodies (Figure 2B). 0.04% of human serum, which is in the linear region of the binding curve, was incubated on a cardiolipin-coated surface in the presence of anti-β2GPI antibodies and the inhibitors. In the presence of anti-β2GPI antibodies, the dimeric molecule, A1-A1, inhibited the binding to cardiolipin of β2GPI in serum much stronger than monomeric A1. The half-maximal inhibition in the presence of anti-β2GPI antibodies was achieved at 10±2 µM of A1-A1 and 218±21 µM of A1. Also, we measured how A1-A1 and A1 inhibited the binding of β2GPI in serum in the absence of anti-β2GPI antibodies (Figure 3). A 1% solution of human serum was titrated with A1-A1 or A1. β2GPI bound to cardiolipin was subsequently detected with anti-β2GPI antibodies. In the absence of anti-β2GPI antibodies, both A1-A1 and A1 were equally ineffective in inhibition of β2GPI. The concentration of the inhibitors at 50% inhibition of β2GPI was 189±34 µM for A1-A1 and 176±37 µM for A1. In sum, the efficiency of A1-A1 to inhibit the binding of β2GPI in serum to cardiolipin was significantly stronger in the presence of anti-β2GPI antibody than in the absence of antibodies. The inhibition efficiency of the monomeric A1 was practically the same and weak regardless of the presence or absence of anti-β2GPI antibodies. A1-A1 was more effective than A1 in inhibition of β2GPI in serum in the presence of anti-β2GPI antibodies.

**Figure 4:**
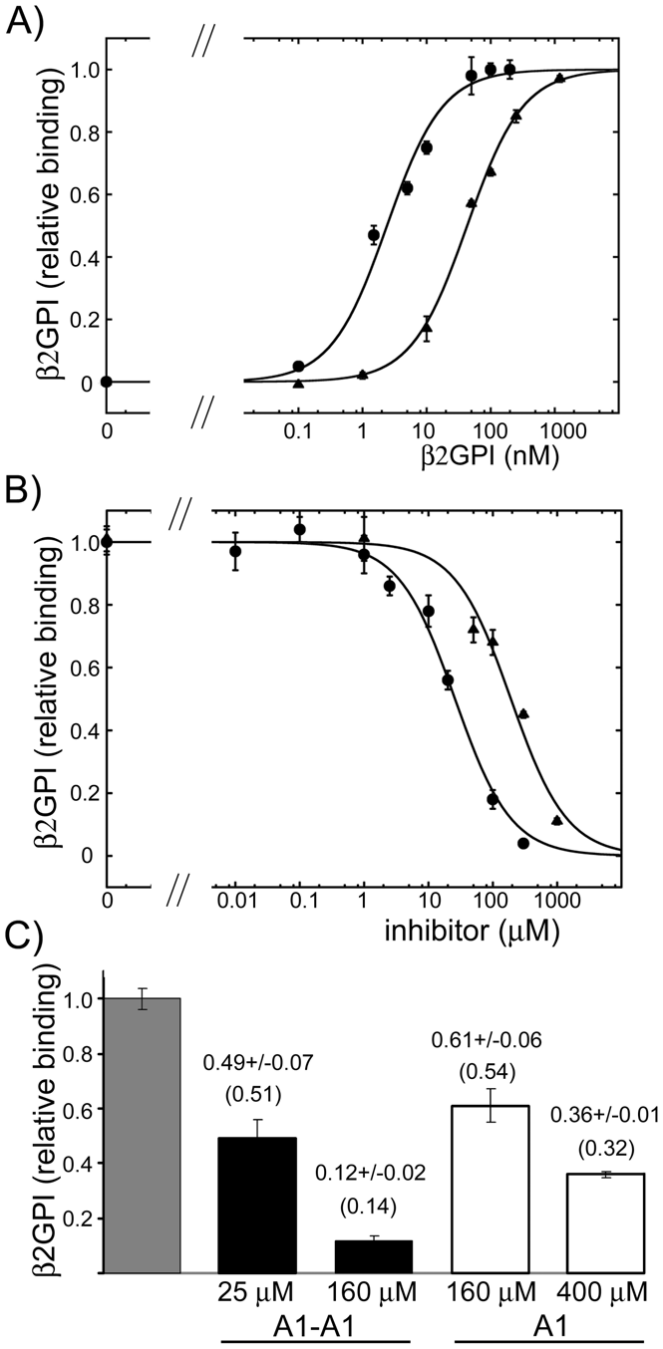
Binding of purified β2GPI to cardiolipin. A) The binding of β2GPI purified from human plasma to cardiolipin in the absence (triangles) and in the presence (circles) of anti-β2GPI antibodies. B) Inhibition of the binding of purified β2GPI to cardiolipin in the presence of anti-β2GPI antibodies by the dimeric inhibitor A1-A1 (circles) and monomeric inhibitor A1 (triangles). Fit to one-site binding and inhibition models was performed on the raw data. The measured OD_405_ values and the binding curves were normalized to the maximum binding obtained from the fit. C) Comparison of the measured with expected binding of β2GPI to cardiolipin calculated based on the fit of the inhibition curves. Purified β2GPI bound to cardiolipin in the presence of anti-β2GPI antibody without inhibitor (gray bar), with A1-A1 (black bars) and with A1 (white bars). The OD_405_ values were normalized to OD_405_ measured in the absence of inhibitor. The values of measured relative binding and standard deviations (±SD) are indicated above bar. The values of expected relative binding were calculated from the fit of the titration data and are given in parenthesis.

**Figure 5:**
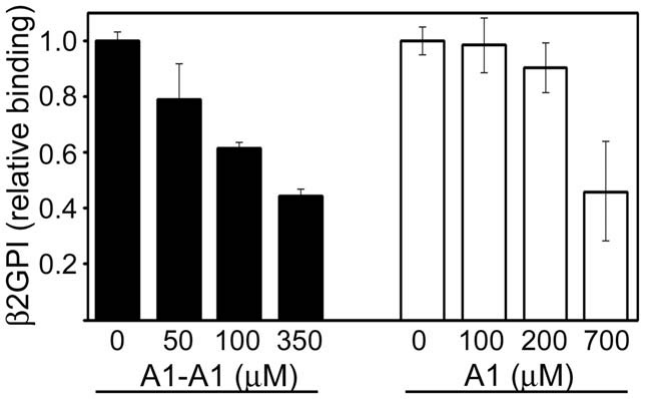
Inhibition of the binding of purified β2GPI to cardiolipin by A1-A1 and A1 in the absence of anti-β2GPI antibodies. The purified β2GPI bound to cardiolipin in the absence of anti-β2GPI antibody with increasing amounts of A1-A1 (black bars) and A1 (white bars). The OD_405_ values were normalized to OD_405_ measured in the absence of inhibitor.

Next, we analyzed how A1-A1 and A1 inhibit the binding of purified β2GPI to cardiolipin. Closed and extended conformations of β2GPI can be interconverted by altering of pH and concentrations of NaCl in the buffer [11], suggesting that the conformation of purified β2GPI may depend on the purification procedure. We used β2GPI purified from human plasma available from Haematologic Technologies, Inc. and analyzed the binding and inhibition of the binding of purified β2GPI by A1-A1 and A1 in the presence and in the absence of anti-β2GPI antibodies. The half-maximal binding was achieved at 2.4±0.4 nM and 43±4 nM of the purified β2GPI in the presence and in the absence of anti-β2GPI antibodies, respectively (Figure 4A). We incubated 10 nM of β2GPI with various concentrations of the dimeric, A1-A1, and monomeric, A1, inhibitors in the presence of anti-β2GPI antibodies. Similarly to what we observed for β2GPI in serum, A1-A1 was more effective in inhibition of the binding of β2GPI to cardiolipin in the presence of anti-β2GPI antibodies. The fit of the titration data to the one-site inhibition model resulted in 26±3 µM of A1-A1 and 191±34 µM of A1 at half-maximal inhibition of purified β2GPI in the presence of anti-β2GPI antibodies (Figure 4B). In a separate experiment, we measured the inhibition of β2GPI in the presence of antibodies and compared the measured values to values predicted by the fit of the titration data (Figure 4C). The measured values were close to those expected from the fit, additionally confirming that in the presence of anti-β2GPI antibodies a much lower concentration of A1-A1 was required to inhibit 50% of the binding of purified β2GPI to cardiolipin compared to A1. In the absence of anti-β2GPI antibodies, both A1-A1 and A1 only weakly inhibited the binding of the purified β2GPI to cardiolipin (Figure 5). Similarly to what we observed for β2GPI in serum, the binding of purified β2GPI to cardiolipin in the presence of anti-β2GPI antibodies was inhibited more strongly by the dimeric inhibitor A1-A1 than by A1. Both, A1-A1 and A1, were ineffective in the inhibition of β2GPI in the absence of anti-β2GPI antibodies.

### Crystal structure of the isolated domain V of β2GPI (β2GPI-DV)

**Figure 6:**
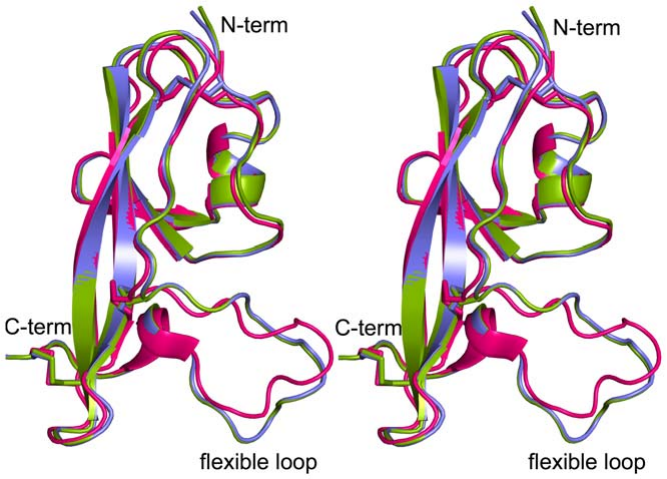
Stereoview of the crystal structure of the isolated domain V of β2GPI (β2GPI-DV). Backbone superposition of the structures of β2GPI-DV chain A (green), chain B (blue) and the crystal structure of domain V from the full-length β2GPI (PDB ID 1C1Z, residues from 244 to 326) (red). The N- and C-termini, and a flexible loop in the domain V are labeled. Figure was generated with the program PYMOL [61]. The two molecules of β2GPI-DV in the asymmetric unit of the crystal, chains A and B, have nearly identical structures with backbone RMSD of 0.23 Å. Backbone superposition of domain V in the full-length β2GPI onto the structure of β2GPI-DV have RMSD of 1.04 Å and 0.97 Å for chains A and B, respectively.

β2GPI binds anionic phospholipids and the A1 modules by its domain V [30], [31], [33], [34], [46]. In the crystal structures of a full-length β2GPI in the extended conformation [8], [9], domain V forms essentially no contacts with the adjacent domain. There are no glycosylation sites in β2GPI that could affect function of domain V indicating that the individual domain V (β2GPI-DV) dissected from the full-length β2GPI will function as domain V in the extended form of β2GPI. We solved the crystal structure of the isolated domain V to 1.9 Å resolution (Table 1). As illustrated by Figure 6, the backbone conformation of β2GPI-DV is nearly identical to the structure of this domain in the full-length β2GPI. The largest difference between the structures is localized to a C-terminal loop. Experimental data strongly suggests that this loop is flexible in the native protein. For example, the residues from 311 to 317 comprising this loop are not defined in one of the crystal structures of the full-length β2GPI (PDB ID 1QUB) [8] and have large values of B-factors in the other (PDB ID 1C1Z) [9]. Also, the residues in the loop are either weak or missing from the NMR spectrum of β2GPI-DV in solution reflecting its internal flexibility [46]. The structural similarity between β2GPI-DV and domain V in the full-length β2GPI provides convincing evidence that the isolated recombinant β2GPI-DV mimics domain V in the extended form of β2GPI.

**Table 1:** Crystallographic statistics.

**Data Statistics**	
Beamline	NSLS X29
Wavelength (Å)	1.075
Space group	P1
Cell parameters (Å)	a = 24.29 b = 38.09 c = 49.51
(°)	α = 93.83 β = 102.65 γ = 90.09
Resolution range (Å) a	38.0–1.9 (2.0–1.9)
Total number of observations^a^	48247 (7052)
Total number of unique^a^	12817 (1850)
Completeness (%)^a^	94.3 (93.8)
I/I(σ) a	12.6 (4.5)
Multiplicity^a^	3.8 (3.8)
R_merge_ (%)^a^	6.1 (26.7)
Molecules in asymmetric unit	2
**Refinement Statistics**	
Free reflections (%)	5
R_work_ (%)	18
R_free_ (%)	21.8
Protein atoms including H	2812
Waters	110
**RMSD from Ideal Geometry**	
Bond angles (°)	1.5
Bond lengths (Å)	0.015
Chirality	0.102
Planarity	0.007
Dihedral	13.4
**Ramachandran Plot**	
number of residues in:	
Preferred regions	159 (96.95%)
Allowed regions	5 (3.05%)
Disallowed regions	0

a Values in parenthesis correspond to the highest resolution shell.

### Comparison of the dimeric inhibitor A1-A1 with monomeric A1. Inhibition of the binding of the isolated domain V of β2GPI (β2GPI-DV) to cardiolipin in the presence of the dimerization antibodies

To analyze how A1-A1 and A1 inhibit the binding of the extended form of β2GPI to cardiolipin, we used purified domain V of β2GPI (β2GPI-DV). We introduced a peptide tag at the N-terminus of β2GPI-DV and used an antibody directed to the tag to form dimeric β2GPI-DV/antibody complexes. We have previously demonstrated that the A1 module binds to the C-terminal part of β2GPI-DV [46] and, therefore, the N-terminal peptide tag on β2GPI-DV and the bound anti-tag antibody will not interfere with the binding of the A1 modules to β2GPI-DV.

**Figure 7:**
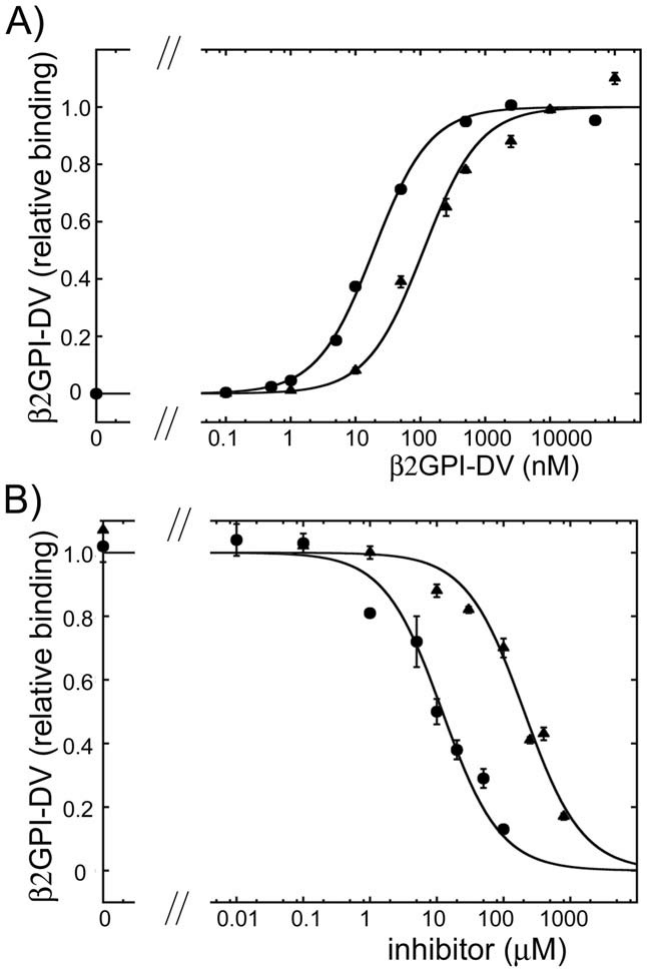
Binding of the individual domain V of β2GPI (β2GPI-DV) to cardiolipin. A) The binding of β2GPI-DV to cardiolipin in the absence (triangles) and in the presence (circles) of the dimerization antibodies. B) Inhibition of the binding of β2GPI-DV to cardiolipin in the presence of dimerization antibodies by the dimeric inhibitor A1-A1 (circles) and monomeric inhibitor A1 (triangles). Fit to one-site binding and inhibition models was performed on the raw data. The measured OD_405_ values and the binding and inhibition curves were normalized to the maximum binding obtained from the fit.

As in the case of the full-length β2GPI, the presence of divalent β2GPI-DV/antibody complexes increased the attachment of β2GPI-DV to cardiolipin (Figure 7A). The fit of the binding data to a one-site model resulted in 19±1 nM of β2GPI-DV and 112±21 nM of β2GPI-DV in the presence and in the absence of anti-tag antibodies. When 30 nM of β2GPI-DV in the presence of anti-tag antibody was incubated with the inhibitors, the half-maximal inhibition was reached at 12±2 µM of A1-A1 and 204±33 µM of A1 (Figure 7B). As we observed for the inhibition of the binding of β2GPI in human serum and purified β2GPI to cardiolipin-coated surfaces, the isolated domain V was inhibited much stronger by the dimeric inhibitor A1-A1 compared to monomeric A1 in the presence of dimeric β2GPI-DV/anti-tag antibody complexes.

### Comparison of β2GPI in human serum with the isolated domain V of β2GPI (β2GPI-DV). Inhibition of the binding to cardiolipin by a monomeric A1 in the absence of antibodies

**Figure 8:**
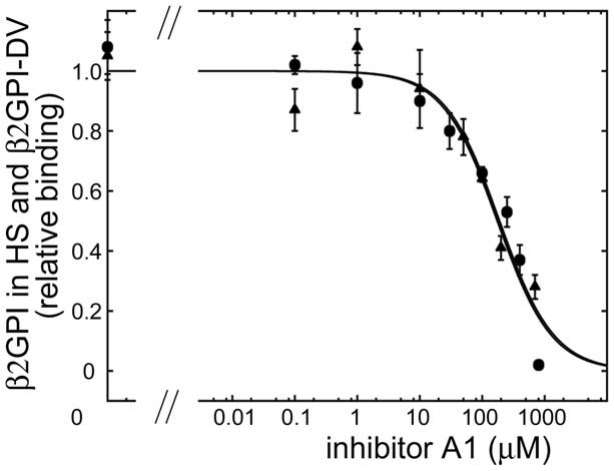
Comparison of the inhibition of β2GPI in serum with the inhibition of the isolated domain V by monomeric A1 in the absence of dimerization antibodies. Inhibition of the binding of β2GPI-DV by A1 (circles); inhibition of the binding of β2GPI in serum by A1 (triangles).

We investigated if the binding of two forms of β2GPI, circular and extended, to cardiolipin is inhibited similarly by A1. We analyzed the inhibition of the monomeric molecules, β2GPI in serum and the isolated domain V, by monomeric A1. The majority of β2GPI in normal human serum is in a circular conformation [11]. The isolated domain V mimics this domain in the extended form of β2GPI. The same concentration of A1 was required to inhibit 50% of the binding of β2GPI in human serum and the individual domain V of β2GPI (Figure 8). The concentration of A1 at half-maximal inhibition was 176±37 µM for β2GPI in serum and 188±44 µM for domain V. This observation demonstrates that A1 binds circular and extended β2GPI with the same affinity suggesting that the binding site for A1 is not obscured in the circular form of β2GPI.

### Stability of the A1-A1 inhibitor in human serum at 37°C

**Figure 9:**
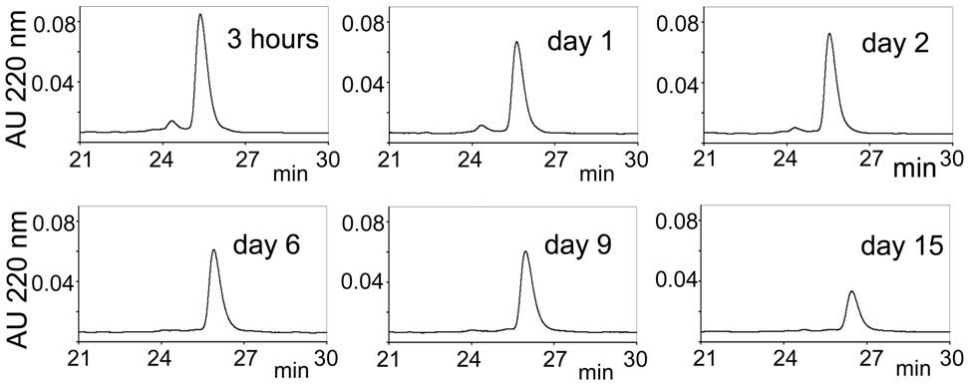
Stability of the A1-A1 inhibitor in human serum at 37°C. HPLC chromatograms of A1-A1 incubated in human serum for the amount of time indicated on each panel. Elution of the A1-A1 inhibitor was monitored with a linear gradient of 0.1% per minute of acetonitrile with 0.1% TFA. The gradient started at 15 minutes from about 26% of acetonitrile/TFA.

To evaluate the susceptibility of the A1-A1 inhibitor to degradation by serum proteases, we incubated A1-A1 in serum at 37°C. Degradation of A1-A1 was monitored by the reversed-phase HPLC by comparing the peak corresponding to the intact A1-A1 on chromatograms collected at different time intervals. The amount of A1-A1 that remained in serum was calculated from the area under the eluted peak. More than 35% of A1-A1 remained in serum after 15 days of incubation at 37°C, indicating that A1-A1 has a favorable stability in serum (Figure 9).

## Discussion

The work reported here examines the effectiveness of a novel dimeric inhibitor A1-A1 to interfere with the binding of pathological β2GPI/anti-β2GPI antibody complexes to anionic phospholipids compared to monomeric A1. The dimeric inhibitor, A1-A1, consists of two ligand-binding A1 modules from ApoER2 connected by a flexible peptide linker. Biophysical characterization of A1-A1 by reverse-phased chromatography confirmed that it is correctly folded in a calcium-dependent manner. Recently, we determined that the bound A1 module prevents the association of β2GPI with anionic phospholipids [46]. Present studies confirmed our previous observations suggesting that the dimeric A1 inhibitor interferes with two pathologically important interactions: the binding of β2GPI/antibody complexes to anionic phospholipids expressed on activated cells and to ApoER2, a lipoprotein receptor on platelets [29], [51].

Normally, β2GPI circulates in the blood plasma as a monomer. Anti-β2GPI antibodies in patients with antiphospholipid syndrome create multivalent β2GPI complexes that have much stronger affinity for anionic phospholipids and lipoprotein receptors than the monomeric β2GPI [44], [52]. Because β2GPI/antibody complexes expressing dimeric β2GPI have prothrombotic properties, in contrast to monomeric pathologically inactive β2GPI, we designed a dimeric inhibitor. We hypothesized that the dimeric molecule A1-A1 preferentially targets multivalent pathological β2GPI/anti-β2GPI antibody complexes leaving monomeric β2GPI, which is abundant in plasma, practically unaffected. To compare A1-A1 and A1 on the inhibition of the binding of β2GPI to anionic phospholipids, we used different preparations of β2GPI, such as β2GPI in normal human serum, β2GPI purified from human plasma and recombinant domain V of β2GPI. β2GPI is a flexible molecule that can adopt a circular [11] and extended conformation [8], [9], [10]. β2GPI in plasma is predominantly in a circular form [11] and the individual domain V closely resembles domain V in the extended conformation of β2GPI, as we demonstrated here by the X-ray crystallography.

We compared the binding curves for two preparations of β2GPI and for β2GPI-DV in the absence and in the presence of the dimerizing antibodies. In all three cases, we observed that the presence of antibodies significantly enhanced the binding of β2GPI and β2GPI-DV to cardiolipin, similarly to what was previously detected for chimeric dimers of β2GPI compared to β2GPI monomers [34]. The binding of β2GPI and β2GPI-DV to cardiolipin-coated surface in the presence of constant amounts of dimerizing antibodies increases with the addition of β2GPI or β2GPI-DV reaching saturation when all antibodies are engaged in complexes. For the inhibition studies, we used concentrations of β2GPI and β2GPI-DV in the linear region of the binding curves at about 50-60% of the maximal binding. Comparison of the binding curves in the presence and in the absence of antibodies suggests that the contribution of β2GPI or β2GPI-DV monomers to total binding in the presence of antibodies is negligible compared to the contribution of β2GPI/antibody or β2GPI-DV/antibody complexes and, therefore, the inhibition curves measured in the presence of antibodies describe the inhibition of a fraction of dimerized molecules.

**Figure 10:**
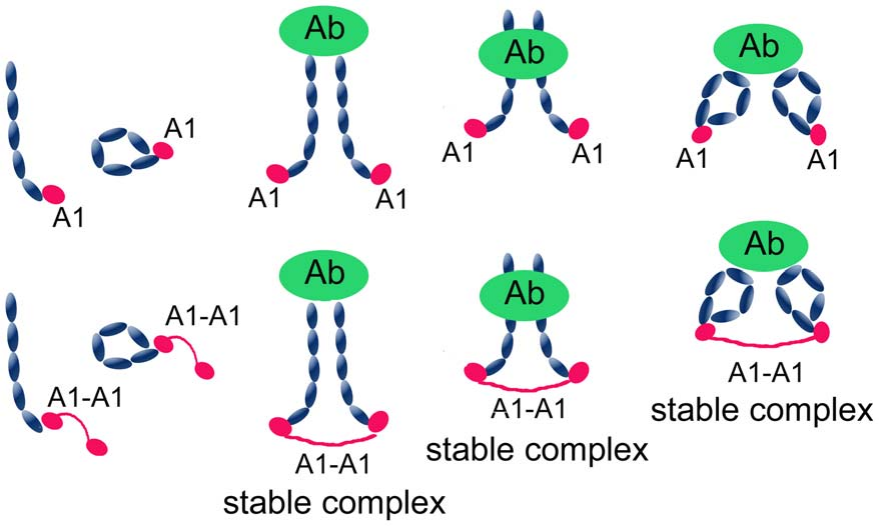
Complex formation between β2GPI, inhibitor and anti-β2GPI antibody. The binding of A1-A1, but not monomeric A1, forms stable β2GPI/anti-β2GPI/A1-A1 complex regardless of localization of the epitope for anti-β2GPI antibody and whether circular or extended β2GPI is dimerized by antibody. β2GPI (blue), A1 or A1-A1 inhibitor (red) and anti-β2GPI antibody (green).

We determined that, regardless of the source of β2GPI, 1) A1-A1 is much more efficient in inhibition of the binding of β2GPI/antibody complexes to anionic phospholipids than A1 and 2) the inhibition of the binding of monomeric β2GPI to anionic phospholipids by either A1-A1 or A1 is practically identical and weak. We also observed that the inhibition of both β2GPI in serum and the individual domain V by A1 is identical in the absence of dimerization antibodies, suggesting that A1 binds the circular and extended forms of β2GPI with the same affinity. Therefore, the binding site for A1 is not obscured on the circular form of β2GPI. Anti-β2GPI antibodies in patients with antiphospholipid syndrome are heterogeneous and their epitopes are scattered over domains I to IV of β2GPI [51], [53]. Some antibodies in patients with antiphospholipid syndrome might bind circular β2GPI and some antibodies might need an extended β2GPI to have their epitopes exposed. Our results demonstrated that when β2GPI, whether circular or extended, is dimerized by anti-β2GPI antibodies, it is more strongly inhibited by A1-A1 than by monomeric A1 by forming stable β2GPI/anti-β2GPI/A1-A1 complexes (Figure 10). We measured in vitro the serum stability of A1-A1. About 35% of A1-A1 remained intact after incubation in serum at 37°C for more than two weeks, indicating that A1-A1 might have favorable pharmacokinetic properties. Given that A1 modules are naturally expressed, the A1-A1 inhibitor is unlikely to be immunogenic.

In our previous work, we have shown that LA modules from different lipoprotein receptors bind to the same site on β2GPI-DV [46]. Therefore, A1-A1 inhibits the binding of β2GPI/antibody complexes not only to ApoER2, but to other lipoprotein receptors as well. Whether other lipoprotein receptors besides ApoER2 contribute to the pathology of antiphospholipid syndrome awaits further investigation. Our results suggest that A1-A1 may be a starting point in the development of the effective inhibitor that interferes with the binding of β2GPI/antibody complexes to anionic phospholipids and lipoprotein receptors. The binding affinity of A1-A1 for β2GPI/anti-β2GPI antibody complexes can be improved in two ways: by optimization of the linker between the two A1 modules and by improving the binding affinity of A1 for β2GPI-DV. Eventually, the A1-based inhibitor can be replaced with small molecule compounds in a dimerized form.

We believe the approach of using a dimeric inhibitor that blocks β2GPI in the pathological multivalent β2GPI/anti-β2GPI complexes holds significant promise. In these studies, we are inhibiting a well characterized binding site for lipoprotein receptors on β2GPI, instead of preventing the binding of antibodies to β2GPI, which are highly heterogeneous in APS patients. Our approach to target the dimerized β2GPI with a dimeric inhibitor could be applied to other pathologically important interactions of β2GPI/antibody complexes. As soon as the binding sites on β2GPI for other APS-related receptors are mapped and characterized in detail, they can be targeted by dimeric inhibitors.

In conclusion, we developed and tested a novel dimeric inhibitor of the β2GPI/antibody complexes. This dimeric inhibitor preferentially targets β2GPI dimerized by anti-β2GPI antibodies compared to pathologically inactive monomeric β2GPI. It prevents the binding of β2GPI/antibody complexes to anionic phospholipids and ApoER2, and might eventually lead to a drug specific for antiphospholipid syndrome.

## Materials and Methods

### Protein expression and purification

Monomeric A1 is a fragment of mouse ApoER2 (residues 12–47 from the mature protein). The dimeric inhibitor, A1-A1, was constructed to contain two A1 fragments connected by a Gly-Ser-Ser-Gly linker. A1 and A1-A1 containing an extra N-terminal Ala and C-terminal Glu-Ala residues were expressed in E.coli as TrpLE fusion proteins and purified from inclusion bodies essentially as previously described [54]. Domain V of β2GPI (residues 244–326), was subcloned into a pET15b vector (Novagen). The encoded protein has an N-terminal histidine tag followed by the sequence recognized by the Tobacco Etch Virus (TEV) protease so that the tag can be removed. To make the domain V of β2GPI recognized by antibodies directed to an HA peptide, the HA sequence, YPYDVPDYA, was introduced at the N-terminus of domain V right after the TEV cleavage site. Domain V with and without the peptide tag was expressed in E.coli, recovered from inclusion bodies, cleaved with TEV and refolded by dialysis at 4°C under conditions permitting disulfide exchange before final purification by reversed-phase HPLC on a C18 column. Protein concentrations were calculated from the measured absorbance of samples at 280 nm using extinction coefficients from the output of ExPASy Protparam tool (http://expasy.org/tools/protparam.html). A full-length β2GPI was purchased from Haematologic Technologies, Inc. Concentrations of β2GPI were calculated using an extinction coefficient at 280 nm E^1%^ of 10 and molecular weight of 54200, as suggested by the supplier.

### Crystallization, data collection and structure determination of β2GPI-DV

Initial crystallization condition was determined in crystallization screen performed at the Hauptman-Woodward Medical Research Institute [55]. The best crystal of β2GPI-DV was obtained at room temperature in hanging drop by combining 1 µL of β2GPI-DV (7 mg/ml in 20 mM HEPES, pH 7.0) with 1 µL of reservoir solution containing 100 mM ammonium sulfate, 40% PEG 1500, 100 mM bis-Tris, pH 7.2. The reservoir solution supplemented with 20% glycerol was used as cryoprotectant. Data was collected from a single crystal at beamline X29A of Brookhaven National Laboratories (NSLS). The crystals belong to the space group P1 with two molecules of β2GPI-DV per asymmetric unit and a solvent content of 45%. Data was processed with MOSFLM [56]. A total of 5% of reflections were excluded and used for R_free_ calculations. The structure was solved by molecular replacement with PHASER [57] using coordinates of domain V extracted from the crystal structure of β2GPI (PDB ID 1C1Z). The initial model determined by PHASER was adjusted with the program COOT [58] and refined using the program REFMAC5 [59]. The final refinement was performed with PHENIX software suit [60].

### Assay for the binding and inhibition of the binding of β2GPI and β2GPI-DV to a cardiolipin-coated surface

Cardiolipin-coated 96 well plates from the ImmunoWELL cardiolipin IgG test kit (GenBio) were blocked with 0.5% of skim milk and 2% BSA in 20 mM Tris, 100 mM NaCl, 2 mM CaCl2, pH 7.4. The assay buffer contained 20 mM Tris, 100 mM NaCl, 2 mM CaCl2, pH 7.4 with 2% BSA and the wash buffer was 20 mM Tris, 100 mM NaCl, 2 mM CaCl2, pH 7.4. When the purified β2GPI (Haematologic Technologies, Inc.) was used in experiments, 27 mM glycine was added to the assay buffer to account for glycine present in the stock solution of β2GPI. β2GPI bound to cardiolipin was detected with peroxidase-conjugated anti-β2GPI antibodies (Cedarlane, CL20021HP, 2 mg/ml) diluted 1∶2500. To detect β2GPI-DV bound to cardiolipin, we used peroxidase-conjugated anti-HA antibody (Abcam, ab1265, 1 mg/ml) directed to HA epitope tag at the N-terminus of β2GPI-DV diluted 1∶2500. The peroxidase activity of the bound antibodies was detected using 2-2′-azino-di-[3-ethylbenzthizzoline] sulfonate (ABTS) chromogenic reagent by measuring OD at 405 nm. All measurements were done in triplicates and corrected to blank before data fitting. The blank contained all components except for β2GPI, serum or β2GPI-DV. The binding and inhibition data was fitted to one-site models using the nonlinear least-squares Marquardt-Levenberg algorithm implemented in GNUPLOT program. The fits of the raw data and the titration data points were then normalized to the maximum binding determined from the fit to facilitate comparison.

For the binding studies, 50 µl of increasing concentrations of either β2GPI (Haematologic Technologies, Inc.), pooled normal human serum (Innovative Research) or the purified recombinant β2GPI-DV were applied to wells and incubated for 1 hour at room temperature. After washing, anti-β2GPI or anti-HA antibody was added to wells and incubated for 1 hour at room temperature before detection. In the second set of experiments, samples containing various concentrations of β2GPI, pooled normal human serum or β2GPI-DV were first incubated for 1 hour at room temperature with the anti-β2GPI or anti-HA antibodies. Then, the samples were applied to cardiolipin, incubated for 1 hour, washed, and bound β2GPI or β2GPI-DV was detected.

For the inhibition studies, increasing concentrations of A1 or A1-A1 were added to a constant amount of β2GPI (50 nM), normal human serum (1%) or β2GPI-DV (130 nM) and incubated for 1 hour at room temperature. Then, 50 µl of the mixtures were incubated on wells for the additional 1 hour. After washing, 50 µl of either anti-β2GPI or anti-HA antibody was added to wells and incubated for 1 hour before detection. In the second set of experiments, 50 µl of samples containing increasing concentrations of A1 or A1-A1 and the constant amounts of either β2GPI (10 nM), normal human serum (0.04%) or β2GPI-DV (30 nM) were first incubated for 1 hour at room temperature with the anti-β2GPI or anti-HA antibodies. Then, samples were incubated on wells for an additional 1 hour and, after washing, the bound β2GPI/anti-β2GPI or β2GPI-DV/anti-tag antibody complexes were detected.

### Measurements of the stability of A1-A1 in serum

Lyophilized A1-A1 purified by reversed-phase chromatography was dissolved in water and its concentration measured by absorbance at 280 nm. The required amount of A1-A1 (180 µg) was then lyophilized and, subsequently, dissolved in 360 µl of pooled normal human serum (Innovative Research). Serum with A1-A1 was filtered through a 0.2 µm eppendorf centrifuge filter, divided into 40 µl samples and set for incubation at 37°C. At timed intervals, 900 µl of 10% acetonitrile with 0.1% TFA in water (buffer A) was added to a 40 µl sample of A1-A1 in serum. Filtered samples were analyzed by reversed-phase HPLC on a C18 column using a linear gradient of 0.1% per minute of buffer B (acetonitrile with 0.1% TFA) staring at 15 minutes from 26% of acetonitrile and monitored for 30 minutes.
